# Periscope

**Published:** 1890-09

**Authors:** 


### PERISCOPE.

### MEDICINE.

**Polyorromenitis, or "Concato's Disease."**—We had feared that the flowers which bloom in the spring would have come and gone, dissipating their fragrance and disarticulating their petals beneath the arid sun of July, without the announcement of a new disease. Our fears were groundless, however, thanks to the rich pathological resources of Italy and the keen observations of Dr. Concato, of Genoa. In the *Rivista clinica e therepeutica*, of February, 1890, this author describes a series of cases, presenting, as he thinks, new and peculiar symptoms. The patients suffer from a progressive and malignant inflammation of various serous membranes, the disease finally assuming the aspect of a severe type of pneumonia. In one case, for example, there was a peritonitis, double pleuritis, and beginning pericarditis. Death occurred on the thirteenth day. The autopsy showed fresh adhesions between the pleural surfaces, with fibrinous exudates in the form of pseudo-membranes above, and of a solid precipitated mass at the base. In the other cases, of which there were five, there was also found fibrinous exudates in the pleural cavity, with evidences, less marked, of peritonitis and pericarditis. The peculiarity of the cases was the disproportion between the severity of the symptoms and the post-mortem findings, which were mainly those of a dry pleurisy. Aside from absence of sputum the cases resembled, clinically, those of pneumonia.

A bacteriological study of the cases by Dr. Bozzolo has given as yet negative results.

In justice to Dr. Concato it should be said that he did not propose the name of "Concato's Disease," but suggests the more scientific one of "polyorromenitis," which is interpreted as meaning a "phthisis of serous membranes." Dr. Galvani Ercoles, however, patriotically prefers to call this Italian trouble "Concato's Disease."

There will always be enough doctors to supply all new diseases with distinctive proper names; but we sometimes fear that this richness in onymic material is being drawn upon too freely. It takes a little practice to say "polyorromenitis" without stumbling, but with proper self-restraint and considerable patience it can be done; and if the esteemed Signor Concato has really found a new disease, we should recommend to systematic writers and teachers practice with the Greek derivative rather than a timorous yielding to the eulaliac ease of pronouncing the perhaps immortal name of Concato.—*Medical Record*, August 2nd, 1890.

L. M. G.

### THERAPEUTICS.

**Intestinal Antisepsis.**—The subject of intestinal antisepsis is not new to the readers of this journal, nor, we trust, to the profession in general. Its importance, however, is certainly not fully appreciated, if one may judge from the absence of all reference to it in recent discussions on the disease—typhoid fever—in which it is most surely indicated. This would be pardonable if intestinal antisepsis, instead of being at the command of every practitioner, were an ideal to be attained by the therapeutics of the future.

The literature on this important subject is mostly of foreign birth or extraction, and the leaders of medical thought in this country, with few exceptions, seem indisposed to admit what is in Europe generally regarded as an accomplished fact.

No one can deny that the intestine is a species of laboratory in which are manufactured poisons of the most virulent nature, and that the chief barrier against their fatal invasion of the general circulation is the liver. This barrier may, however, be forced, and this happens when the liver is overcome by an excess of leucomaines, or, on account of functional disorder—*hepatic inadequacy*, so to speak—is unable to dispose of a normal invoice of these products.

These facts are no sooner stated than acknowledged; indeed they are property common to the layman and the physician. The association between such signs of systematic poisoning as ill-humour, hypochondria, and constipation, is proverbial, and no one has been more keenly appreciative of the fact than Voltaire, who terminates an admirable description of the good nature and benevolence of those whose bowels are regular with the statement that a refusal from the mouth of such a person is more gracious than an assent from one who is constipated.

In a recent article on the treatment of diarrhœa and constipation by Dujardin-Beaumetz (*Bulletin Génèrale de Thèrapeutique*, April 15th, 1890), the importance of intestinal antisepsis in both these conditions is insisted upon. The fact is not generally appreciated that purgation and antisepsis are, to some extent, interchangeable terms, and that the relief which follows the employment of a cathartic is not so much due to the mechanical effect of "unloading the bowels" as to the expulsion of substances which are slowly poisoning the system. These substances not only act in the manner just indicated, but through their irritating effect on the intestine they tend to keep up a diarrhœa which, in its onset, may have been highly conservative. From these facts may be explained the curative effect of a purge in the early stages of some forms of diarrhœa. When, however, a catarrhal condition is fairly established and occupies a considerable portion of the intestinal tract, the indications are not fully met by cathartics. Under such circumstances admirable results are often obtained by drugs whose favourable action can only be explained by intestinal antisepsis. Among such are thymol, naphthol, and salicylate of bismuth. There is none better than the first mentioned, which is perfectly innocuous in large doses and possesses an antiseptic power four times greater than that of carbolic acid. Its value as an antiseptic seems to have been more fully recognised in Italy than elsewhere, and has been endorsed by Martini, Bufalini, Testi, and others. In our opinion it is, when properly administered and in suitable doses, *facile princeps* among intestinal antiseptics. Salicylate of bismuth is also an admirable remedy of this sort, and is the one most approved by Dujardin-Beaumetz in the article mentioned. He usually administers it in capsules in combination with magnesia, sodium bicarbonate, prepared chalk, phosphate of lime, beta-naphthol, or charcoal.

It is not for want of suitable drugs that intestinal antisepsis is not more systematically carried out. The difficulty is that the indications for their employment are not generally recognised. Systematic therapeutics of any description is the outcome of systematic diagnosis, and the latter, so far as gastro-intestinal affections are concerned, is of very recent date.

There are, as Dujardin-Beaumetz points out, at least three distinct varieties of auto-intoxication proceeding from the gastro-intestinal tract. First, there are the various symptoms, too numerous to be here detailed, dependent upon habitual constipation. Secondly, a more acute condition, of which the chief symptoms are a coated tongue, loss of appetite, and sometimes fever. This is the *embarras gastrique* of the French, and is caused by the ingestion of improper food, or of "*toxines*" contained in the atmosphere. The latter statement is based on the fact that the symptoms referred to often arise after a prolonged stay in a poorly ventilated and overcrowded apartment, or in a dissecting-room. The third variety is accompanied with vomiting, and is generally known in this country as acute indigestion. It is significant of more acute poisoning or of a more susceptible organism, and consequently is more frequently observed in children.

The treatment of these different forms of poisoning, whether it be exclusively dietetic or partly medicinal, is fundamentally antiseptic, and the general recognition of this fact will alone serve to bring into general use a line of treatment which is now habitually followed by a minority.—*Medical News*, June 7th, 1890.

L. M. G.

**Mercury during Albuminuria.**—The question as to whether mercury is likely to be injurious in cases in which albumen is present in the urine, is one of much importance to the surgeon as well as the physician. Many surgical complications occur to patients who are the subjects of chronic renal disease, and conditions are often presented in which, were there not a prejudice against it, mercury would seem to be desirable. I have myself chiefly been concerned with this dilemma in cases of syphilis, and have long been in the habit of prescribing mercury without much regard to the state of urine. Nor have I ever seen reason to regret doing so. The following quotation from a Report on Medicine by the late Dr. Prichard, of Bristol (1835), is not without its interest in reference to this question. Dr. Prichard refers to cases in which dropsy was the condition requiring treatment.

"It seems to have been the opinion of Dr. Bright, and the observation was strenuously enforced by Dr. Blackall, that mercury is injurious in all cases of this description. I shall take the liberty to state that this question has been brought to the test of experiment, during several years, at the Bristol Infirmary. In numerous cases of dropsy, with albuminous urine, the treatment advised by Dr. Blackall has been fully tried, and it has failed to produce a cure; but the same cases terminated in recovery under a moderate use of mercurial remedies."—Mr. Jonathan Hutchinson in *Archives of Surgery*, July, 1890.

